# Increased airway resistance can be related to the decrease in the functional capacity in obese women

**DOI:** 10.1371/journal.pone.0267546

**Published:** 2022-06-07

**Authors:** Larissa Perossi, Mayara Holtz, Daniele Oliveira dos Santos, Jéssica Perossi, Hugo Celso Dutra de Souza, Wilson Salgado Junior, Ada Clarice Gastaldi

**Affiliations:** 1 Health Sciences Department, Ribeirão Preto Medical School, University of São Paulo, Ribeirão Preto, São Paulo, Brazil; 2 Surgery Department, Ribeirão Preto Medical School, University of São Paulo, Ribeirão Preto, São Paulo, Bazil; Universita degli Studi di Milano, ITALY

## Abstract

**Background and objective:**

Obesity can increase the airways resistance, mainly in the periphery, leading to dyspnea perception that can impair the functional capacity. This study aimed to analyze if airways resistance could be related to the walking capacity of women with morbid obesity.

**Methods:**

Thirty-seven women with grade III obesity in preoperative bariatric surgery were evaluated using the spirometry test, impulse oscillometry system (IOS), and six-minute walk test (6MWT). Additionally, data about their daily dyspnea perception and physical activity level were collected.

**Results:**

Variables of the spirometry test did not detect ventilator disorders. Compared to the predicted values, the IOS identified significant increase in airways resistance (kPa/L/s) (R5: 0.36 (0.34; 0.36) and 0.53 (0.47; 0.61); R20: 0.30 (0.28; 0.30) and 0.41 (0.35; 0.45); R5-20: 0.06 (0.06; 0.06) and 0.14 (0.10; 0.15); X5: -0.03 (-0.04; -0.01) and -0.20 (-0.27; -0.18), respectively). The distance walked in the 6MWT, 491.4±60.4m was significantly correlated to R5 (rho = -0.41, *p* = 0.01), R5-20 (rho = -0.52, p = 0.001), and X5 (rho = 0.54, p = 0.0006).

**Conclusion:**

The IOS is able to identify changes in airway resistance even before the onset of symptoms. When evaluated by IOS women with severe obesity and normal spirometry exhibited central and peripheral airways obstruction. The correlations between the IOS and six-minute walk distance suggest that increased peripheral airways resistance could be related to worsening functional capacity.

## Introduction

The increase in obesity prevalence represents a public health problem, and the degree of obesity is directly related to morbidity and mortality risks [1]. Several functional consequences can be found in obese individuals due to the deposition of adipose tissue on the abdomen and around the ribcage. These alterations impose several effects on the respiratory system leading to structural changes (lower diaphragmatic and thorax mobility) that cause mechanical alterations (respiratory compliance and resistance alterations) and biochemical dysfunctions (airways inflammation) [2].

Structural changes result in decrease of pulmonary volumes and capacities, mainly in functional residual capacity (FRC) and the expiratory reserve volume (ERV), inducing an increased peripheral airways resistance that may lead to dyspnea. Dyspnea is a common symptom in obese individuals and may be related to weight gain, either in the absence of respiratory diseases or associated with other comorbidities that can affect airway obstruction and gas exchange but, the causes are not completely understood [2, 3].

Spirometry is the gold standard test for lung function evaluation in clinical practice. However, the findings regarding the type of alteration (restrictive or obstructive) in the lung function of grade III obesity patients are still controversial [4, 5]. Restrictive changes occur due to the decrease in pulmonary compliance and the obstructive disorders occur due to collapsed small airways [5]. However, the spirometry test does not have the sensitivity to detect peripheral airways disease in severe obese subjects [6–8].

Previous studies with obese individuals without respiratory symptoms using the Impulse Oscillometry System (IOS) found peripheral airway dysfunctions while their spirometry test was normal [6, 7, 9]. The IOS has been considered a sensitive test to detect early airways impedance alterations, especially those in the periphery. The respiratory system impedance comprises the assessment of resistance and reactance that can predict small airways disease even without clinical signs or symptoms, allowing interventions to be performed as soon as possible to avoid severe pulmonary complications [9–11].

Thus, IOS can identify the respiratory system resistance in a compartmentalized way, while the spirometry test has the limitation to detect in which lung region there are some airway obstruction [11]. Therefore, this study aimed to analyze if airways resistance could be related to walking capacity in grade III obesity women.

## Methods

### Participants

This cross-sectional study was conducted in an outpatient clinic of a university hospital. The volunteers were recruited from March 2015 to October 2016 after approval of the local Research Ethics Committee (CAAE n°34717314.5.0000.5440). All volunteers were aware about the study procedures and signed the informed consent form.

The sample size was calculated for R5 based on the results of Albuquerque et al., with an effect size of 0.13 Kpa/L/s, a standard deviation of 0.17, α of 5%, and power of 90, resulting in 36 participants [6].

The inclusion criteria were female, non-smokers, with BMI ≥ 40kg/m^2^, and age between 18 and 50 years. Individuals with chronic obstructive pulmonary disease, asthma, or restrictive pulmonary diseases; obstructive sleep apnea; cardiovascular, musculoskeletal, and/or neuromuscular diseases; uncontrolled diabetes; middle ear disorders; retinal glaucoma; and abdominal hernias were excluded from the study.

### Procedures

The evaluations were conducted at the Laboratory for Assessment of Respiratory System following the order of the sub items below.

The evaluation of central and peripheral airways resistance was performed by the IOS technique [10, 11]. The Jaeger Impulse Oscillation System (Jaeger, Wurzburg, Germany) was used to evaluate respiratory system impedance, which comprises the assessment of airflow resistance and reactance by the application of pressure pulses in multiple frequency ranges of sound waves over the respiratory system during spontaneous breathing. The test was conducted through breathing in a circular mouthpiece coupled with a free flow piece according to the method suggested by Oosteven et al [10]. The total airway resistance (R5), central airway resistance (R20), peripheral airways resistance (R5-R20) and reactance at 5Hz (X5) were calculated with frequencies of 5 to 20 Hz. The equation used to compare the obtained values with those predicted was developed by Vogel & Smith that included individuals with age between 18 and 69 years old [12].

The spirometry test (Koko PFT System; version 4.11, 2007 nSpire Health Inc.; Pulmonary Data Services, United States) was performed to detect the presence of any respiratory disorder which could be an exclusion criterion. For the pulmonary function tests, we followed the methodology described in the Brazilian Guidelines for Pulmonary Function Tests [13]. The equation proposed by Pereira et al. was used to calculate the predicted values of variables. This equation was developed for Brazilian population between 20 and 85 years [14]. The variables analyzed were: forced vital capacity (FVC), forced expiratory volume in one second (FEV_1_), FEV_1_/FVC and mean forced expiratory flow (FEF_25–75%_). The percentage of predicted values was calculated to eliminate the age-related confounding factor and was expressed as %FVC, %FEV_1_ and %FEF_25-75%_.

The six-minute walk test (6MWT) was performed according to the methodology described in the ATS/ERS Statement and the equation suggested by Soares et al. was used to obtain the predicted distance (6MWD) [15, 16]. The heart rate (HR), peripheral oxygen saturation (SpO_2_), respiratory rate (RR), blood pressure (BP) and the modified Borg scale (for dyspnea and fatigue in lower limbs) were monitored at rest, at the third minute during the test, immediately after the test (6^th^ minute), and at the third (Recovery 1) and sixth (Recovery 2) minutes after the test [17].

The mMRC was used to evaluate the dyspnea perception of the participants. This scale has 5 items that correspond to the subjective value for breathlessness during exercise: 0 (during vigorous exercise), 1 (when walking briskly or ascending a gentle slope), 2 (walking slower than other people with the same age or having to stop when walking slowly), 3 (stopping after walking after 100 meters or after a few minutes) and 4 (severe dyspnea that makes it impossible to leave home or when getting dressed) [18].

The short version of the International Physical Activity Questionnaire (IPAQ) was used to evaluate the minutes of physical activity performed during one week, including activities performed for leisure, sport, exercise, or as part of their activities at home. According to the score of the questionnaire, the participants were classified as very active, active, irregularly active or sedentary [19].

### Statistical analysis

The software R Core Team (version 3.4.3, Vienna, Austria, 2017) and the software GraphPad Prism 6.0 (GraphPad Software, San Diego, California) were used. Data distribution was tested using the Shapiro-Wilk test. The Student’s t-test and Wilcoxon test were used to compare predicted and obtained values. The Spearman Correlation test was used to perform the correlations. The results were considered significant with *p* < 0.05.

## Results

Thirty-seven participants were recruited, and their anthropometric and demographic characteristics are shown in Table 1. The spirometry test did not detect obstruction. The 6MWD was 491.4±60.4 and there was no significant difference when compared to the predicted values (Table 2).

**Table 1:** Anthropometric and demographic data of the participants.

**Age (years)**	37 (22, 50)
**Body weight (kg)**	119.5 (97.3, 183.0)
BMI (kg/m^2^)	45.1 (40.0, 62.8)
**W/HR**	0.87 (0.75, 1.08)

Results expressed as median (min, max)

Abbreviations: BMI: body mass index; W/HR: waist–hip ratio

**Table 2:** Spirometric data and 6MWD (absolute and predicted values) of the participants.

	Predicted values	Obtained values	% Predicted
**FVC (L)**	3.64±0.39	3.50±0.72	95.11±12.80
**FEV_1_ (L)**	3.02±0.31	2.85±0.59	93.72±14.14
**VEF_1_/CVF**	0.83±0.01	0.81±0.05	97.76±6.84
**FEF_25-75%_ (L/min)**	3.55±0.18	3.07±0.96	87.03±29.02
**6MWD (m)**	487.1±40.0	491.4±60.4	101.0±10.8

Results are expressed as mean±SD

Abbreviations: FVC: forced vital capacity; FEV_1_: forced expiratory volume in one second; FEV_1_/FVC: Tiffenau index; FEF_25-75%_: mean forced expiratory flow; 6MWD: six-minute walk distance

**Fig 1:**
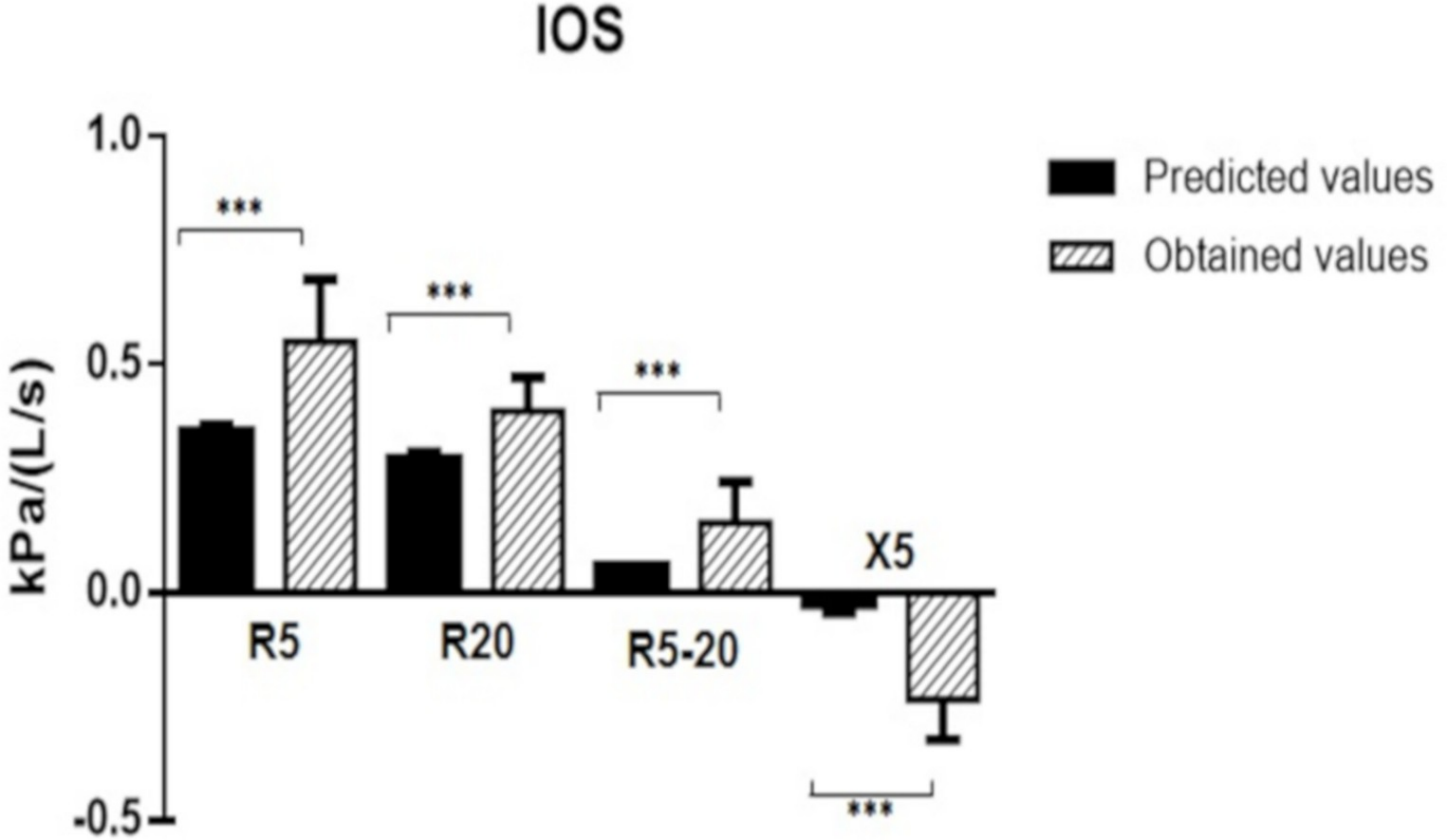
Impulse oscillometry measurements. Comparison of resistance at an oscillation frequency of 5 and 20 Hz (R5, R20, R5-20) and reactance at 5 Hz (X5) between the predicted and obtained values. *** p < 0.05.

The IOS assessment showed increased resistance (Kpa/L/s) in central and peripheral airways as compared with the predicted values, R5: 0.53 (0.47; 0.61) and 0.36 (0.34; 0.36); R20: 0.41 (0.35; 0.45) and 0.30 (0.28; 0.30); R5-20: 0.14 (0.10; 0.15) and 0.06 (0.06; 0.06), respectively. Further, the reactance (Kpa/L/s) at 5Hz was significantly more negative, X5: -0.20 (-0.27; -0.18) and -0.03 (-0.04; -0.01), respectively (Fig 1).

About mMRC, 29 (78,4%) participants reported breathless during intense exercise (mMRC = 0), or walking briskly or ascending a gentle slope (mMRC = 1). The IPAQ classified 22 (59,5%) participants as active and 8 (21,6%) as very active.

**Fig 2:**
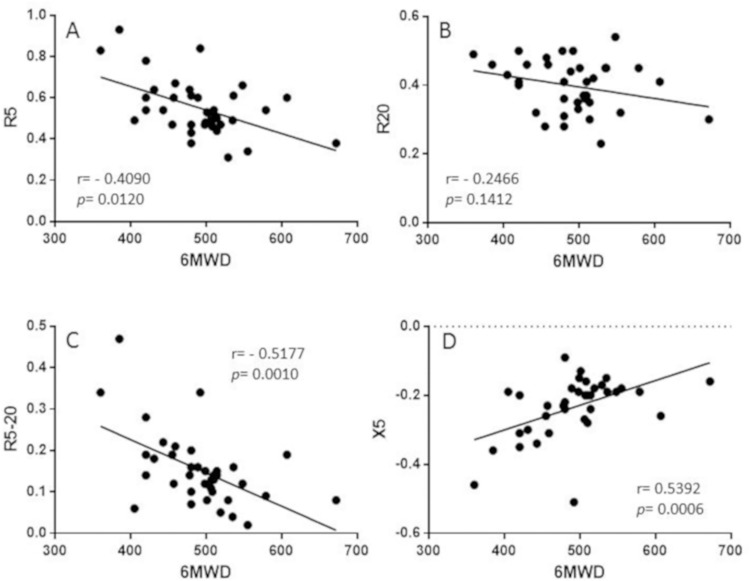
Correlation between impulse oscillometry and 6-minute walk distance (6MWD). A: correlation between R5 and 6MWD, B: correlation between R20 and 6MWD, C: correlation between R5-20 and 6MWD, D: correlation between X5 and 6MWD.

The 6MWD was significantly correlated with R5 (rho = -0.4090; *p* = 0.0120), R5-20 (rho = -0.5177; *p* = 0.0010), and X5 (rho = 0.5392; *p =* 0.0006). The correlation between 6MWD and R20 was not significant (rho = -0.2466; *p* = 0.1412) (Fig 2).

## Discussion

This study analyzed the correlation of functional capacity and airways resistance in grade III obesity women. The correlation between the 6MWD and IOS parameters showed that increased resistance is related to the decrease in the walk distance and, until now, we have not found studies that have evaluated similar correlation between 6MWD and airways resistance. According to our hypothesis, in participants who did not exhibit any disorders in spirometry, the IOS was able to detect increased respiratory system resistance, indicating central and peripheral airways obstruction [11]. In contrast to our expectations; most of the participants had no complaints of dyspnea, were physically active and had a walking capacity within the normal range. Despite this, resistance and reactance at 5 Hz was significant correlated the walking test, the data showed moderate correlation, suggested that impairment of peripheral resistance may modify the performance in the 6MWD.

Our study evaluated only female subjects due to the greater number of women in the bariatric surgery clinics of local service. Besides the higher prevalence of obese women in worldwide and Brazil (15% and 24,4%, respectively), it is possible to identify in studies that evaluated pulmonary function and IOS parameters this same prevalence [6, 8, 9, 20–22]. And, in order to compare the predicted values of spirometry and oscillometry, reference equations are different between genders because women tend to have a greater central and peripheral resistance than men due to height and diameter of the bronchi [11, 14]. Thus, it is expected that, besides the higher airways resistance in women and, obesity can further aggravate this condition for men or women.

Some changes in the respiratory system of obese subjects may be closely related to the excess of adipose tissue around the chest wall, abdomen, and upper airways, which may cause a reduction in lung volume that can contribute to structural changes that affect the respiratory system resistance [23, 24]. Brazzalle et al. reported that spirometry evaluation is important to confirm obstructive changes in the respiratory physiology of obese individuals and, Melo et al. concluded that most of the obese subjects are more susceptible to develop a restrictive pattern, while some studies have shown spirometry results in the normal range in obese patients [3, 9, 22–24]. These studies and our finds confirm the finding that spirometry may not be as sensitive for diagnosing peripheral airway disorders.

Thus, our study also corroborates with the effectiveness of IOS. While our participants did not present dysfunctions in the spirometry test and did not report dyspnea symptoms, it was detected an early increase in their respiratory system resistance. According to other studies, our results showed an increase in the central and peripheral airways in obese subjects [7, 9]. The study of Albuquerque et al. found similar values for central and peripheral airways resistance for the grade III obesity individuals [6]. However, they did not found difference of central resistance when compared to the control group. Despite these findings, none of these studies evaluated the dyspnea perception in obese people.

The mechanism of small airways disease in obese subjects is not completely clear and can be also related to inflammation by the action of the substances released by the adipose tissue that culminates with the expression of pro-inflammatory markers (TNFα, IL-1β e IL-6), however Van de Kant et al. did not detect the presence of airway inflammation based on the fraction of exhaled nitric oxide and, further studies are needed to confirm data related to airway inflammation in obese patients [7].

The increased peripheral airways resistance may be related to perceived dyspnea, which is often found in individuals with Grade II and III obesity, but that was not identified in the present study [3]. Two studies in COPD patients did not find significant correlation between perceived dyspnea and airways resistance by IOS [25, 26]. However, we know that dyspnea is multifactorial and that the respiratory limitations in obese individuals and those with COPD are not comparable.

The distance walked by our patients was similar to that observed in the study of Santarém et al. but, they did not report the physical activity level of the participants [27]. It is known that the walked distance may be reduced in severe obesity individuals by some factors, such as difficulty in walking due to musculoskeletal pain caused by an overload on the lower limbs and by low exercise capacity [28]. Furthermore, the impact of the degree of disability for this group on the 6MWT is influenced by weight, BMI, waist circumference, and flexibility [29]. As a clinical consequence, the functional capacity and the activities of daily living must be also affected and, this condition may be aggravated by sedentary lifestyle, with decreased walking capacity and greater perceived dyspnea [27].

It is interesting to note that, in our study, the participants did not present a decrease in functional capacity on the 6MWT (compared to the predicted distance) or complaints of dyspnea. It can be explained by the management of the multidisciplinary team that assists these patients, encouraging them to practice physical activity regularly and to change their sedentary lifestyle. Moreover, it is important to emphasize that weight is one of the variables included in the 6MWD equation which may be responsible for reducing functional capacity and the lowest predicted distance mainly in grade III obesity individuals [16]. Regarding the variables analyzed in the walking test, we think that the level of physical activity (active and very active) and the absence of respiratory symptoms in activities of daily living may explain the return of cardiopulmonary parameters (HR, RR and BP) close to baseline in the third minute of recovery in 6MWT, even with the impairment in the peripheral airways.

As our results suggested that the involvement of peripheral airways is related to the walked distance, further research is needed to better identify the mechanisms involved in this relationship, especially in sedentary obese individuals who may present more respiratory symptoms.

Considering bariatric surgery as an option for severe obesity subjects, Santiago et al. observed an improvement in FEV_1_ and FVC, even though they were within normal range in the preoperative period, and Peters et al. found a decrease in the central airway resistance at supine position after surgery [8, 22]. Adding to these finds, our study suggests that even in patients who do not present any clinical signs or symptoms, the fragility of the small airways, mainly, can interfere in the intra and/or postoperative periods or long periods of bed restriction. So, it is important to describe that impairment in the airways may difficult mechanical ventilation or extubation, and may increase probability of the small airways collapse. In this way, our recommendation is to maintain a greater attention in the preoperative preparation of the obese individuals who present some alteration in the small airways with the purpose of reducing the complications and optimizing the functional recovery in the postoperative period.

As limitations of our study, we did not collect data about airways inflammation; sedentary participants with dyspnea complaints were not included.

In conclusion, this study suggests that the presence of central and peripheral airways obstruction in severe obesity women that can be related with 6MWT performance. And, the results confirmed that IOS is able to identify changes in airway resistance even before the onset of symptoms.

## Supporting information

S1 Table Anthropometric and demographic data 37 grade III obesity women. BMI: body mass index; W/HR: waist–hip ratio.
(PDF)

S2 Table Spirometric variables of 37 grade III obesity women. Pred: Predicted values. L: Liters. FVC: Forced vital capacity. FEV_1_: Forced expiratory volume in one second. FEV_1_/FVC: Tiffenau index. FEF_25-75%_: Mean forced expiratory flow.
(PDF)

S3 Table Impulse oscillometry variables of 37 grade III obesity women. Pred: Predicted values. R5: Total airways resistance. R20: Central airways resistance. R5-R20: Peripheral airways resistance. X5: Reactance at 5 Hz.
(PDF)

S4 Table mMRC results and Six-Minute Walk Test variables of 37 grade III obesity women. mMRC: modified Medical Research Council. 6MWT: Six-minute walk test.
(PDF)

## References

[1] World Health Organization. Obesity: preventing and managing the global epidemic. Report of a World Health Organization Consultation. Geneva: World Health Organization, 2000. p. 256. WHO Obesity Technical Report Series, n. 284. 11234459

[2] Sebastian JC. Respiratory physiology and pulmonary complications in obesity. Best Pract Clin Endocrinol Metab. 2013; 27: 157–161. doi: 10.1016/j.beem.2013.04.014 23731878

[3] Teixeira CA, dos Santos JE, Silva GA, de Souza EST, Baddini-Martinez JA. [Prevalence of and he potential pathophysiological mechanisms involved in dyspnea in individuals with class II or III obesity]. J Bras Pneumol. 2007; 33: 28–35. doi: 10.1590/s1806-37132007000100008 17568865

[4] Parameswaran K, Todd DC, Soth M. Altered respiratory physiology in obesity. Can Respir J. 2006; 13: 203–210. doi: 10.1155/2006/834786 16779465 PMC2683280

[5] Rabec C, de Lucas Ramos P, Veale D. Respiratory complications of obesity. Arch Bronconeumol. 2011; 47: 252–261. doi: 10.1016/j.arbres.2011.01.012 21458904

[6] Albuquerque CG, Andrade FM, Rocha MA, et al. Determining respiratory system resistance and reactance by impulse oscillometry in obese individuals. J Bras Pneumol. 2015; 41: 422–426. doi: 10.1590/S1806-37132015000004517 26578133 PMC4635088

[7] van de Kant KD, Paredi P, Meah S, Kalsi HS, Barnes PJ, Usmani OS. The effect of body weight on distal airway function and airway inflammation. Obes Res Clin Pract. 2016; 10: 564–573. doi: 10.1016/j.orcp.2015.10.005 26620577

[8] Peters U, Hernandez P, Dechman G, Ellsmere J, Maksym G. Early detection changes in lung mechanics with oscillometry following bariatric surgery in severe obesity. Appl Physiol Nutr Metab. 2016; 41: 1–10. 27109263 10.1139/apnm-2015-0473

[9] Oppenheimer BW, Macht R, Goldring RM, Stabile A, Berger KI, Parikh M. Distal airway dysfunction in obese subjects corrects after bariatric surgery. Surg Obes Relat Dis. 2012; 8: 582–589. doi: 10.1016/j.soard.2011.08.004 21955746

[10] Oostveen E, MacLeod D, Lorino H, et al. The forced oscillation technique in clinical practice: methodology, recommendations and future developments. Eur Respir J, 2003; 22: 1026–1041. doi: 10.1183/09031936.03.00089403 14680096

[11] Brashier B, Salvi S. Measuring lung function using sound waves: role of the forced oscillation technique and impulse oscillometry system. Breathe. 2015; 11: 57–65. doi: 10.1183/20734735.020514 26306104 PMC4487383

[12] Vogel J, Smidt U. Impulse oscillometry: analysis of lung mechanics in general practice and clinic, epidemiological and experimental research. Frankfurt: PMI-Verlagsgruppe; 1994.

[13] Brazilian Society of Pneumology and Tisiology. [Brazilian Guidelines for Pulmonary Function Tests]. J Bras Pneumol. 2002; 28: S1–S82.

[14] Pereira CAC, Sato T, Rodrigues SC. [New reference values for forced spirometry in white adults in Brazil.] J Bras Pneumol. 2007; 33: 397–406. doi: 10.1590/s1806-37132007000400008 17982531

[15] Holland AE, Spruit MA, Troosters T, et al. An official European Respiratory Society/American Thoracic Society technical standard: field walking tests in chronic respiratory disease. Eur Respir J. 2014; 44: 1428–1446. doi: 10.1183/09031936.00150314 25359355

[16] Soares MR, Pereira CAC. [Six-Minute Walk Test: reference values for healthy adults in Brazil]. J Bras Pneumol. 2011; 37: 576–583. doi: 10.1590/s1806-37132011000500003 22042388

[17] Kendrick KR, Baxi SC, Smith RM. Usefulness of the modified 0–10 Borg scale in assessing the degree of dyspnea in patients with COPD and asthma. J Emerg Nurs. 2000; 26: 216–222. doi: 10.1016/s0099-1767(00)90093-x 10839848

[18] Kovelis D, Segretti NO, Probst VS, Lareau SC, Brunetto AF, Pitta F. [Validation of Modified Pulmonary Functional Status and Dyspnea Questionnaire and Medical Research Council for patients with chronic obstructive pulmonary disease in Brazil]. J Bras Pneumol. 2008; 34: 1008–1018. doi: 10.1590/s1806-37132008001200005 19180335

[19] Matsudo S, Araújo T, Matsudo V, et al. [International Physical Activity Questionnaire (IPAQ): Validity and reliability study in Brazil]. Atividade física e saúde. 2001; 6: 5–18.

[20] World Health Organization [Internet]. Obesity and overweight [cited 2018 Nov 13]. Available from http://www.who.int/news-room/fact-sheets/detail/obesity-and-overweight.

[21] Health Ministry [Internet]. [More than half of adults are overweight] [cited 2018 Nov 13]. Available from: http://www.brasil.gov.br/noticias/saude/2015/08/mais-da-metade-dos-adultos-estao-acima-do-peso.

[22] Santiago A, Carpio C, Caballero P, et al. [Effect of weight loss after bariatric surgery on respiratory function and sleep apnea-hypopnea syndrome in women with morbid obesity]. Nutr Hosp. 2015; 32: 1050–1055. doi: 10.3305/nh.2015.32.3.9487 26319819

[23] Melo LC, da Silva MAM, Calles ACN. [Obesity and pulmonary function: a systematic review]. Einstein (São Paulo), 2014 Jan-Mar; 12: 120–125. 24728258 10.1590/S1679-45082014RW2691 PMC4898251

[24] Brazzale DJ, Pretto JJ, Schachter LM. Optimizing respiratory function assessments to elucidate the impact of obesity on respiratory health. Respirology, 2015; 20: 715–721. doi: 10.1111/resp.12563 26033636

[25] Anderson WJ, Lipworh BJ. Relationships between impulse oscillometry, spirometry and dyspnoea in COPD. J R Coll Physicians Edinb, 2012; 42: 111–115. doi: 10.4997/JRCPE.2012.204 22693693

[26] Aarli BB, Calverley PMA, Jensen RL, Eagan TML, Bakke PS, Hardie JA. Variability of within-breath reactance in COPD patients and its associations with dyspnoea. Eur Respir J, 2015; 45: 625–634. doi: 10.1183/09031936.00051214 25359342

[27] Santarém GCF, de Cleva R, Santo MA, et al. Correlation between body composition and walking capacity in severe obesity. *PloS One* 2015 Jun 22; 10: e0130268. doi: 10.1371/journal.pone.0130268 26098769 PMC4476574

[28] Anandacoomarasamy A, Caterson ISambrook, Fransen M, March L. The impact of obesity on the musculoskeletal system. Int J Obes, 2008; 32: 211–222. doi: 10.1038/sj.ijo.0803715 17848940

[29] de Souza SA, Faintuch J, Fabris SM, et al. Six-minute walk test: functional capacity of severely obese before and after bariatric surgery. Surg Obes Relat Dis, 2009; 5: 540–543. doi: 10.1016/j.soard.2009.05.003 19656738

