# Supplementary material for: Increased airway resistance can be related to the decrease in the functional capacity in obese women
Source: PLoS One. 2022 Jun 7;17(6):e0267546. doi: 10.1371/journal.pone.0267546 (PMC9173605; doi:10.1371/journal.pone.0267546)
Supplement: S1 Table — BMI: body mass index; W/HR: waist–hip ratio. (PDF) [file pone.0267546.s001.pdf]

Table S1. Anthropometric and demographic data 37 grade III obesity women

Code	Age	Weight (kg)	Height (cm)	BMI	W/HR
O01	32	154,7	157	62,8	0,89
O02	33	104,9	162	40,0	0,79
O03	34	101,8	157	41,3	0,87
O04	29	135,8	172	45,9	0,93
O05	37	105,3	156	43,3	0,87
O06	29	106,2	163	40,0	0,76
O07	41	112	159	44,3	0,84
O08	32	106,4	158	42,6	0,93
O09	42	100,7	157	40,9	0,75
O10	44	133,2	165	48,9	0,85
O11	43	119,5	165	43,9	0,86
O12	42	122	156	50,1	0,88
O13	37	113,6	159	44,9	0,83
O14	44	145	164	53,9	0,87
O15	42	114,1	162	43,5	0,85
O16	50	115	165	42,2	0,89
O17	22	131,8	176	42,5	0,93
O18	28	160	166	58,1	0,93
O19	34	132,6	159	52,5	0,91
O20	43	139,1	162	53,0	0,81
O21	50	118,2	149	53,2	0,77
O22	27	129,9	171	44,4	0,88
O23	28	144	170	49,8	0,79
O24	41	144,4	155	60,1	0,92
O25	39	149	179	46,5	0,86
O26	34	109,3	160	42,7	0,90
O27	44	119	156	48,9	0,90
O18	34	107	165	39,3	0,79
O19	33	105,9	158	42,4	0,93
O30	23	150,1	168	53,2	0,80
O31	28	183	175	59,8	0,84
O32	42	133,5	167	47,9	0,99
O33	24	124	165	45,5	0,96
O34	48	125,9	167	45,1	1,06
O35	25	125,5	159	49,6	0,91
O36	44	102	159	40,3	1,08
O37	38	97,3	153	41,6	0,87

BMI: body mass index; W/HR: waist–hip ratio
