# Supplementary material for: Increased airway resistance can be related to the decrease in the functional capacity in obese women
Source: PLoS One. 2022 Jun 7;17(6):e0267546. doi: 10.1371/journal.pone.0267546 (PMC9173605; doi:10.1371/journal.pone.0267546)
Supplement: S2 Table — Pred: Predicted values. L: Liters. FVC: Forced vital capacity. FEV1: Forced expiratory volume in one second. FEV1/FVC: Tiffenau index. FEF25-75%: Mean forced expiratory flow. (PDF) [file pone.0267546.s002.pdf]

Table S2. Spirometric variables of 37 grade III obesity women.

Code	FVC pred	FVC (L)	FVC (%)	FEV1 pred	FEV1 (L)	FEV1 (%)	FEV1/FVC pred	FEV1/FVC	FEV1/FVC (%)	FEF25-75 pred	FEF25-75 (L/min)	FEF25-75 (%)
O01	3,67	2,64	72	2,9	2,24	77	0,79	0,85	107	3,39	2,72	80
O02	3,63	3,42	94	2,87	2,63	92	0,79	0,77	97	3,37	2,17	64
O03	3,47	3,77	109	2,74	3,3	121	0,79	0,88	111	3,27	4,72	144
O04	4,23	4,69	111	3,56	3,7	104	0,85	0,79	93	4	3,49	87
O05	3,22	3,32	103	2,78	2,77	100	0,86	0,83	97	3,37	3,07	91
O06	3,84	3,51	91	3,05	3,01	99	0,79	0,86	108	3,53	4,28	121
O07	NA	NA	NA	NA	NA	NA	NA	NA	NA	NA	NA	NA
O08	3,48	3,6	104	3,01	3,14	104	0,87	0,87	101	3,64	4,01	124
O09	3,19	3,63	114	2,69	2,87	107	0,84	0,79	94	3,12	2,66	85
O10	3,61	3,09	86	2,98	2,58	87	0,82	0,83	102	3,22	2,99	109
O11	3,26	3,21	98	2,75	2,45	89	0,84	0,76	91	3,18	1,97	62
O12	3,21	3,27	102	2,48	2,73	110	0,77	0,83	108	2,97	3,11	105
O13	NA	NA	NA	NA	NA	NA	NA	NA	NA	NA	NA	NA
O14	3,6	3,26	90	2,99	2,62	87	0,83	0,80	97	3,29	2,53	77
O15	3,51	3,61	103	2,93	3,11	106	0,83	0,86	104	3,26	3,91	120
O16	3,28	2,68	82	2,69	2,3	86	0,81	0,86	106	2,88	3,16	110
O17	4,51	5,1	113	3,82	3,99	105	0,85	0,78	92	4,32	3,71	86
O18	3,91	3,63	93	3,35	2,77	83	0,86	0,76	89	3,94	2,26	57
O19	3,48	3,81	109	2,99	3,39	113	0,86	0,89	104	3,57	4,62	130
O20	3,17	3,12	98	2,72	2,66	98	0,86	0,85	99	3,48	3,11	89
O21	2,58	1,7	66	2,17	1,17	54	0,83	0,69	83	2,54	0,62	24
O22	4,2	4,25	101	3,56	3,65	103	0,85	0,86	101	4,03	4,7	117
O23	4,08	4,24	104	3,46	3,08	89	0,85	0,73	85	3,95	2,25	57
O24	3,28	2,96	90	2,78	2,32	83	0,84	0,78	93	3,23	2,05	64
O25	4,41	4,51	102	3,58	3,31	92	0,8	0,73	91	3,66	2,46	67
O26	3,51	3,42	97	3	2,58	86	0,85	0,75	88	3,54	2,03	57
O27	3,12	1,91	61	2,64	1,54	58	0,84	0,81	96	3,06	1,5	49
O18	3,78	3,59	95	3,2	2,98	93	0,85	0,83	98	3,66	3,26	89
O19	3,63	3,31	91	3,1	2,92	94	0,85	0,88	103	3,61	4,17	115
O30	4,06	3,98	98	3,51	3,24	92	0,87	0,81	93	4,18	3,51	84
O31	4,4	4,44	101	3,69	3,72	101	0,84	0,83	99	4,09	3,89	95
O32	3,7	3,49	94	3,06	3	98	0,82	0,86	105	3,32	3,83	115
O33	4,06	4,19	103	3,51	3,46	99	0,87	0,83	95	4,18	3,53	85
O34	3,62	3,92	108	2,94	3,16	107	0,81	0,81	100	3,06	3,07	100
O35	4,3	2,43	57	3,74	2,18	58	0,86	0,89	103	4,41	3,07	70
O36	3,27	2,93	90	2,74	2,47	90	0,83	0,84	101	3,11	2,88	93
O37	3,1	2,7	87	2,69	2,17	81	0,86	0,8	93	3,29	2,07	63

Pred: Predicted values. L: Liters.FVC: Forced vital capacity. FEV_1: Forced expiratory volume in one second. FEV_1/FVC: Tiffenau index. $FEF_{25-75\%}$: Mean forced expiratory flow.
