# Supplementary material for: Increased airway resistance can be related to the decrease in the functional capacity in obese women
Source: PLoS One. 2022 Jun 7;17(6):e0267546. doi: 10.1371/journal.pone.0267546 (PMC9173605; doi:10.1371/journal.pone.0267546)
Supplement: S3 Table — Pred: Predicted values. R5: Total airways resistance. R20: Central airways resistance. R5-R20: Peripheral airways resistance. X5: Reactance at 5 Hz. (PDF) [file pone.0267546.s003.pdf]

Table S3. Impulse oscillometry variables of 37 grade III obesity women

Code	R5 pred	R5	R20 pred	R20	R5-R20 pred	R5-R20	X5 pred	X5
O01	0,35	0,83	0,29	0,49	0,06	0,34	-0,02	-0,46
O02	0,35	0,6	0,29	0,41	0,06	0,19	-0,02	-0,31
O03	0,35	0,38	0,29	0,28	0,06	0,1	-0,02	-0,24
O04	0,34	0,66	0,28	0,54	0,06	0,12	-0,01	-0,19
O05	0,36	0,49	0,3	0,43	0,06	0,06	-0,03	-0,19
O06	0,34	0,61	0,28	0,41	0,06	0,2	-0,01	-0,22
O07	0,36	0,54	0,3	0,4	0,06	0,14	-0,03	-0,2
O08	0,35	0,49	0,29	0,45	0,06	0,04	-0,02	-0,15
O09	0,36	0,47	0,3	0,28	0,06	0,19	-0,04	-0,26
O10	0,37	0,47	0,31	0,31	0,06	0,16	-0,04	-0,09
O11	0,36	0,67	0,3	0,46	0,06	0,21	-0,04	-0,31
O12	0,36	0,48	0,3	0,37	0,06	0,11	-0,04	-0,27
O13	0,36	0,43	0,3	0,36	0,06	0,07	-0,03	-0,22
O14	0,37	0,6	0,31	0,48	0,06	0,12	-0,04	-0,23
O15	0,36	0,47	0,3	0,42	0,06	0,05	-0,04	-0,18
O16	0,38	0,54	0,32	0,32	0,06	0,22	-0,06	-0,34
O17	0,33	0,53	0,27	0,45	0,06	0,08	0,01	-0,13
O18	0,34	0,5	0,28	0,35	0,06	0,15	-0,01	-0,24
O19	0,35	0,54	0,29	0,41	0,06	0,13	-0,02	-0,2
O20	0,37	0,78	0,31	0,5	0,06	0,28	-0,04	-0,35
O21	0,38	0,93	0,32	0,46	0,06	0,47	-0,07	-0,36
O22	0,34	0,34	0,28	0,32	0,06	0,02	0	-0,18
O23	0,34	0,5	0,28	0,37	0,06	0,13	-0,01	-0,2
O24	0,36	0,64	0,3	0,46	0,06	0,18	-0,04	-0,3
O25	0,36	0,6	0,3	0,41	0,06	0,19	-0,03	-0,26
O26	0,35	0,61	0,29	0,45	0,06	0,16	-0,02	-0,19
O27	0,37	0,84	0,31	0,5	0,06	0,34	-0,04	-0,51
O18	0,35	0,38	0,29	0,3	0,06	0,08	-0,02	-0,16
O19	0,35	0,54	0,29	0,45	0,06	0,09	-0,02	-0,19
O30	0,33	0,6	0,27	0,44	0,06	0,16	0,01	-0,18
O31	0,34	0,48	0,28	0,33	0,06	0,15	-0,01	-0,15
O32	0,36	0,64	0,3	0,5	0,06	0,14	-0,04	-0,23
O33	0,33	0,46	0,27	0,36	0,06	0,1	0	-0,16
O34	0,37	0,47	0,31	0,35	0,06	0,12	-0,05	-0,19
O35	0,34	0,44	0,28	0,3	0,06	0,14	0	-0,2
O36	0,37	0,31	0,31	0,23	0,06	0,08	-0,04	-0,17
O37	0,36	0,51	0,3	0,37	0,06	0,14	-0,03	-0,28

Pred: Predicted values. R5: Total airways resistance. R20: Central airways resistance. R5-R20: Peripheral airways resistance. X5: Reactance at 5 Hz.
