# Supplementary material for: Increased airway resistance can be related to the decrease in the functional capacity in obese women
Source: PLoS One. 2022 Jun 7;17(6):e0267546. doi: 10.1371/journal.pone.0267546 (PMC9173605; doi:10.1371/journal.pone.0267546)
Supplement: S4 Table — mMRC: modified Medical Research Council. 6MWT: Six-minute walk test. (PDF) [file pone.0267546.s004.pdf]

Table S4. mMRC results and Six-Minute Walk Test variables of 37 grade III obesity women.

	mMRC					6MWT		
**Code**	**0**	**1**	**2**	**3**	**4**	**Predicted distance**	**Walked distance**	**%predicted**
O01			X			425	360	84,7
O02	X					536	420	78,4
O03	X					521	480	92,1
O04		X				519	548	105,6
O05	X					501	405	80,8
O06	X					555	480	86,5
O07		X				482	420	87,1
O08		X				523	535	102,3
O09		X				496	455	91,7
O10		X				450	480	106,7
O11		X				478	459	96,0
O12	X					451	506	112,2
O13		X				493	480	97,4
O14			X			426	457	107,2
O15		X				483	519	107,5
O16	X					465	443	95,2
O17		X				566	501	88,5
O18		X				464	514	110,8
O19			X			467	510	109,2
O20					X	433	420	97,0
O21	X					404	385	95,3
O22		X				534	555	103,9
O23		X				504	507	100,6
O24			X			405	431	106,4
O25		X				478	607	127,0
O26	X					515	536	104,1
O27	X					448	492	109,8
O18		X				531	672	126,6
O19	X					521	579	111,1
O30			X			489	489	100,0
O31	X					449	499	111,1
O32			X			486	478	98,4
O33	X					532	508	95,5
O34		X				487	498	102,3
O35			X			492	514	104,5
O36	X					509	529	103,9
O37	X					505	510	101,0

mMRC: modified Medical Research Council. 6MWT: Six-minute walk test
